# Obesity, Bioactive Lipids, and Adipose Tissue Inflammation in Insulin Resistance

**DOI:** 10.3390/nu12051305

**Published:** 2020-05-03

**Authors:** Iwona Kojta, Marta Chacińska, Agnieszka Błachnio-Zabielska

**Affiliations:** Department of Hygiene, Epidemiology and Metabolic Disorders, Medical University of Bialystok, Jana Kilińskiego 1, 15-089 Bialystok, Poland; paszkiewicziwona89@gmail.com (I.K.); marta.chacinska@gmail.com (M.C.)

**Keywords:** obesity, insulin resistance, adipose tissue, biologically active lipids, adipokines, cytokines

## Abstract

Obesity is a major risk factor for the development of insulin resistance and type 2 diabetes. The exact mechanism by which adipose tissue induces insulin resistance is still unclear. It has been demonstrated that obesity is associated with the adipocyte dysfunction, macrophage infiltration, and low-grade inflammation, which probably contributes to the induction of insulin resistance. Adipose tissue synthesizes and secretes numerous bioactive molecules, namely adipokines and cytokines, which affect the metabolism of both lipids and glucose. Disorders in the synthesis of adipokines and cytokines that occur in obesity lead to changes in lipid and carbohydrates metabolism and, as a consequence, may lead to insulin resistance and type 2 diabetes. Obesity is also associated with the accumulation of lipids. A special group of lipids that are able to regulate the activity of intracellular enzymes are biologically active lipids: long-chain acyl-CoAs, ceramides, and diacylglycerols. According to the latest data, the accumulation of these lipids in adipocytes is probably related to the development of insulin resistance. Recent studies indicate that the accumulation of biologically active lipids in adipose tissue may regulate the synthesis/secretion of adipokines and proinflammatory cytokines. Although studies have revealed that inflammation caused by excessive fat accumulation and abnormalities in lipid metabolism can contribute to the development of obesity-related insulin resistance, further research is needed to determine the exact mechanism by which obesity-related insulin resistance is induced.

## 1. Introduction

Epidemiological studies on obesity and overweight demonstrate that these are significant, constantly growing problems that have reached the status of global epidemics. According to the World Health Organization (WHO), the number of obese people has tripled in the last 20 years [1]. The main reasons for the increasing number of people with obesity are mainly improper dietary habits and sedentary lifestyle. Obesity is a state of pathological increase in the amount of adipose tissue, which boosts the risk of numerous diseases, such as cardiovascular disease, some types of cancer, and type 2 diabetes [2,3]. There are a number of causes leading to the development of obesity, including genetic and environmental factors. The contribution of genetic factors to obesity is very important and is thought to be responsible for 40–70% of obesity cases [4,5]. However, it appears that non-genetic factors, especially environmental factors such as unhealthy eating habits and lack of physical activity, also play a substantial role in generating obesity [6]. Studies have shown that not only the number of calories consumed, but also the type of diet and the frequency and timing of meals have an impact on the development of obesity and related metabolic disorders [6,7,8]. In addition, it has been found that independent of diet and physical activity, also sleep duration and daytime sleepiness affect overweight and obesity [9].

Health complications resulting from overweight and obesity have become one of the main causes of mortality in developed countries, making them the fifth most frequent cause of death worldwide. According to the latest WHO reports, the proportion of obese people in the global population has more than doubled since 1980. Today, over 1.9 billion adults are overweight and more than 600 million are obese [10]. Obesity-related adipose tissue mass gain requires further detailed research to understand the influence of this tissue on the induction of many related diseases, including cardiovascular diseases, osteoarthritis, diabetes, and insulin resistance.

Insulin, secreted by β cells located in pancreatic islets, is an anabolic hormone that causes glycogen accumulation in the liver and skeletal muscles. This hormone lowers blood glucose levels by increasing glucose uptake by muscles and adipose tissue, stimulates glucose oxidation and glycogenesis, and inhibits gluconeogenesis and glycogenolysis. It also stimulates lipogenesis and inhibits lipolysis, which leads to the storage of free fatty acids (FFA) in the form of triacylglycerols (TAG) in adipose tissue. Furthermore, it increases the uptake of amino acids by tissues and enhances protein synthesis [11]. Insulin resistance, as defined by the American Diabetes Association (ADA), is a condition in which the response of cells to insulin is impaired with respect to carbohydrates, lipids, and proteins, resulting in elevated blood glucose levels [12]. Insulin has a wide spectrum of effects on metabolic processes in adipocytes; therefore, it is considered the most important hormone regulating anti-lipolytic processes, and deterioration of cell sensitivity to this hormone or impairment of the insulin pathway may affect the metabolism of adipose tissue.

## 2. Type of Obesity Adipose Tissue Localization and Insulin Resistance

In 1947, a French diabetologist Jean Vague discovered the relationship between gender and obesity types, and defined two types of obesity based on the location of adipose tissue: android (abdominal obesity) and gynoid (gluteofemoral obesity). The first type, i.e., the central accumulation of adipose tissue, dominates in men, whereas subcutaneous fat in the hip and thigh regions is more common in women [13]. Android obesity is characterized by the accumulation of both subcutaneous fat in the abdominal region and visceral fat. This type of obesity has been shown to be associated with a three-fold higher risk of cardiovascular disease compared to gynoid obesity [14]. It has also been observed that android type of obesity particularly promotes the development of insulin resistance and type 2 diabetes and is often accompanied by hypertension, dyslipidemia with high levels of TAG and low-density lipoproteins (LDL), and low levels of high-density lipoproteins (HDL). All these factors are components of the metabolic syndrome [15]. Abdominal obesity also contributes to systemic inflammation that is associated with the synthesis and secretion of proinflammatory cytokines [15]. Visceral adipose tissue (VAT) differs from subcutaneous adipose tissue (SAT) not only in terms of morphology, but also functionality. These tissues can have different effects on the induction of lipid and glucose metabolism disorders. This may be because fatty acids released during lipolysis with VAT are supplied directly to the liver through the portal venous system.

Subcutaneous adipose tissue is characterized by a higher number of small adipocytes and higher insulin sensitivity. This tissue has a higher expression of the insulin receptor substrate (IRS-1) as well as a greater ability to activate it. For this reason, the antilipolytic effect of insulin is stronger in the subcutaneous fat tissue [16,17]. On the other hand, the visceral adipose tissue is characterized by a higher number of large adipocytes. This feature is one of the possible risk factors associated with the development of insulin resistance of this tissue, as well as other metabolic diseases [17]. According to the studies conducted thus far, the abdomen subcutaneous fat in terms of lipolytic activity is similar to VAT. Available data indicate that, the greater is the accumulation of subcutaneous fat in the abdominal area, the greater is the similarity of this tissue to visceral tissue. Accordingly, the lipolytic activity of this tissue increases and hence the rate of FFA release [16]. SAT from the gluteal-femoral region has much lower lipolytic activity than VAT. This may be due to the different activity of transcriptional factors that regulate the expression of the enzymes and other proteins implicated in lipid metabolism [18]. Apart from the differences between the said tissues, differences were also observed within the subcutaneous adipose tissue itself. These concern the lipolytic activity of the subcutaneous abdominal adipose tissue compared to the subcutaneous adipose tissue located in the thigh area [19,20]. It has been demonstrated that the lipolytic activity of subcutaneous adipocytes in the gluteal-femoral region is weaker than in the abdominal area, which partly explains smaller impact of this tissue accumulation on the development of metabolic dysfunction. This may be due to the action of sex steroid hormones (estrogen and androgens) [18,21,22,23,24], but this protective effect can also be triggered by adiponectin, synthesized in significant amounts by adipose tissue located in the lower parts of the body. Adiponectin affects the expression of fatty acid transporters and enzymes responsible for fatty acid oxidation, which results in more efficient absorption of circulating fatty acids [25]. This relationship was confirmed by Tanko et al., who showed that the concentration of adiponectin in blood is higher and tissue sensitivity to insulin is more pronounced in obese women with peripheral fat tissue distribution compared to women with visceral obesity [24]. The relationships described above suggest that the subcutaneous gluteal-femoral tissue may reduce the risk of insulin resistance by more effective uptake of circulating fatty acids, thus protecting other organs from excess FFA [26]. Moreover, McLaughlin et al. pointed out that in both men and women with moderate obesity or overweight, elevated VAT mass increases the risk of insulin resistance, while increased SAT mass reduces this risk [27]. In addition, the percentage of VAT was significantly higher in the group of insulin resistant patients compared to the insulin sensitive group, whereas SAT located in the gluteal-femoral region showed an inverse relationship [27].

## 3. Lipid Metabolism in Adipose Tissue

Under physiological conditions, most fat from ingested food (over 95%) is accumulated in adipose tissue and is stored in the form of triglycerides. The metabolism of adipose tissue is affected by: hormonal factors (mainly insulin and catecholamines), autocrine and paracrine factors, nutritional status (food intake and starvation), stressors, and physical activity.

In the conditions of increased energy requirement, e.g., during physical activity, while feeling cold, and in stressful situations such as periods of starvation, the stored TAG are hydrolyzed during the lipolysis process, releasing both FFA and glycerol. Lipolysis is one of the main metabolic processes occurring in adipose tissue. Its main stimulators are catecholamines, growth hormone, glucagon, natriuretic peptides (NP), and TSH [16]. Catecholamines, including adrenaline and noradrenaline, act through adrenergic receptors located in the adipocyte membrane. In humans, an important role in triggering the process of lipolysis through stimulating the adrenergic receptors is played by the receptors β1, β2, and, to a lesser extent, β3, which is most active in rodents [16]. In adipocytes, the binding of β-adrenergic receptor agonists (β1 and β2) conjugated with adenyl cyclase leads to increased production of cyclic adenosine monophosphate (cAMP) and activation of protein kinase A (PKA), which then phosphorylates hormone-sensitive lipase (HSL) on three sites: Ser563, Ser659, and Ser660 [28]. Activation of HSL entails the decomposition of TAG, initiated by triglyceride lipase—an enzyme present in visceral and subcutaneous fat cells—hydrolyzing TAG to diglycerides, and HSL decomposes diglycerides to monoglycerides [29]. An important factor regulating the lipolysis process in adipose tissue is the natriuretic peptide. The mechanism by which NP enhances lipolysis involves activation of guanosine cyclic monophosphate (cGMP) signaling and hormone sensitive lipase translocation [30,31,32]. Clinical studies have shown that, in metabolic diseases such as obesity and T2D, reduced plasma NP levels are observed, which can be used to predict type 2 diabetes [30,31,32]. An increase in the supply of energy substrates in relation to the body’s requirements leads to the accumulation of their excess in adipose cells in lipogenesis process. This process is regulated by insulin, which increases the activity of lipoprotein lipase (LPL) stimulating the hydrolysis of TAGs circulating in plasma in combination with albumins, chylomicrons or very low-density lipoproteins (VLDL), thus allowing FFAs to enter the cell. Such transport is enabled by specific transporters including CD36 protein, fatty acid transport proteins (FATPs) and fatty acid-binding protein plasma membrane (FABPpm). Acyl-CoA synthetase (ACS) is an enzyme that converts FFA to acetyl-CoAs. Then, acyl-CoAs can be a substrate in the de novo synthesis of other lipids including TAG, which are most abundant in adipocytes. In TAG de novo synthesis, an important role is played by the enzyme glycerol-3-phosphate acyltransferase (GPAT) that catalyzes the acylation of glycerol 3-phosphate to produce lysophosphatidic acid (LPA). Subsequently, LPA is converted into phosphatidic acid (PA), Then, PA is dephosphorylated by the action of the enzyme phosphatidic acid phosphatase (PAP) which leads to the formation of diacylglycerols (DAG). The conversion of DAG to TAG is catalyzed by diacylglycerol acyltransferase (DGAT). Glycerol-3-phosphate comes from the metabolism of glucose, which occurs in adipocytes under the influence of insulin [33,34].

As long as adipose tissue is able to store excess energy as a result of hypertrophy (increased size of cells) and/or hyperplasia (increased number of cells), the development of metabolic disorders is limited. However, hypertrophic adipocytes become resistant to the antilipolytic effect of insulin and have a reduced ability to accumulate lipids. Once these adipocyte storage capacities are exceeded, fat accumulates in, inter alia, muscle and liver cells, causing their insulin resistance [35]. In this process, biologically active lipids seem to play an important role, which have been repeatedly indicated as the main factor in inducing insulin resistance of skeletal muscles and the liver [36,37,38,39,40,41,42,43,44].

## 4. Biologically Active Lipids

Obesity is accompanied by increased plasma concentrations of FFA, leading to their intensified uptake by tissues involved in the regulation of glucose homeostasis (skeletal muscles, liver, and pancreas). The source of FFA can be both the subcutaneous and visceral adipose tissue [45]. Due to the higher lipolytic activity of visceral adipocytes, the release of FFA from VAT is faster compared to subcutaneous fat, which was observed in both obese and lean patients [46]. Increased fatty acid uptake, exceeding the cell’s ability to oxidize them in mitochondria, leads to intracellular lipid accumulation. This may result in the development of a phenomenon called lipotoxicity, which favors the reduction of insulin sensitivity and gradual atrophy of β cells in the pancreas. In the past, it was assumed that insulin resistance was induced by triacylglycerols. However, attention is currently focused mainly on biologically active lipids capable of inhibiting or activating enzymes that directly affect the insulin pathway. These lipids include long-chain acyl-CoA (LCACoA) [47,48], ceramides (Cer) [25,36,37,49], and DAG [40,50].

### 4.1. Long Chain Acyl-CoA (LCACoA)

After passing through the cell membrane, fatty acids are activated by attaching coenzyme A (CoA) in a reaction catalyzed by ACS, resulting in the formation of LCACoA. Activated fatty acids, in the form of LCACoA, are substrates in de novo synthesis of other lipids (triacylglycerols, diacylglycerols, and ceramides) or undergo β-oxidation in the mitochondria [51]. It has been demonstrated that LCACoAs activate some isoforms of protein kinase C (PKC), which results in serine phosphorylation at 307 (Ser307) in IRS-1. This activation prevents tyrosine phosphorylation in the insulin receptor and, consequently, the binding and activation of phosphoinositide 3-kinases (PI3K) necessary for proper insulin action [52,53,54].

Literature data indicate that LCACoA levels in obese people increase not only in skeletal muscle or liver, but also in adipose tissue. Studies on fat tissue in obese people demonstrated an increase in the levels of C16:1-CoA, C16-CoA, C18:1-CoA, and C18-CoA in SAT compared to the control group (lean individuals) [55]. Moreover, the total content of LCACoA was higher in obese patients compared to the controls in both subcutaneous and epicardial adipose tissue. In epicardial adipose tissue, a significant correlation was also observed between palmitoyl-CoA (C16-CoA) and HOMA-IR [55].

### 4.2. Diacylglycerols (DAG)

Diacylglycerols are very important second messengers involved in the regulation of intracellular processes such as proliferation, differentiation, and signal transduction in the nervous system. However, their excessive accumulation leads to the activation of certain isoforms of protein kinase C (PKC) that are responsible for abnormal insulin signaling, which leads to impaired GLUT4 translocation to the cell membrane. These compounds may be formed in a cell as a result of the breakdown of phospholipids or in de novo synthesis. The key stage of de novo DAG synthesis is the glycerol-3-phosphate acylation catalyzed by GPAT1. The direct product of this reaction is lysophosphatidic acid. Studies have shown that overexpression of GPAT1 can lead to steatosis and increased insulin resistance [56]. On the other hand, hydrolysis of phosphatidylinositol-4,5-bisphosphate occurs as a result of the action of phospholipase C-β. Intramuscular accumulation of DAG was found in insulin-resistant rats fed high-fat diet [57] as well as Zucker rats [58]. Moreover, a threefold increase in liver DAG content was observed in rats infused with intralipid, resulting in inhibition of the insulin pathway. In vitro studies on isolated skeletal muscle and adipocytes have shown that the activation of specific PKC isoforms by DAG reduces insulin dependent glucose uptake, while pharmacological inhibition of these PKC activity doubles glucose uptake. The observed changes were associated with tyrosine phosphorylation in the IRS-1 insulin receptor as well as with PI3K kinase activity [59]. In addition, a significant positive correlation was observed between total DAG content in the subcutaneous adipose tissue and HOMA-IR [55,60,61]. However, it is still unknown how diacylglycerols contribute to the development of insulin resistance of adipose tissue.

### 4.3. Ceramide (Cer)

Ceramide is the central compound of sphingolipid metabolism. This lipid plays an important role in regulating intracellular processes such as proliferation, differentiation, and apoptosis. It can be formed either by hydrolysis of sphingomyelin located in membranes or by de novo synthesis [62]. The first and key stage of this process is the condensation of serine and palmitoyl-CoA. This reaction is catalyzed by serine palmitoyltransferase (SPT). The direct product of this reaction is 3-ketosphinganine, which is rapidly transformed into sphinganine [63]. Then, another fatty acid is attached to the sphinganine molecule, resulting in the formation of dihydroceramide. This reaction is catalyzed by the enzyme dihydroceramide synthase [64]. Another method of ceramide generation is the so-called salvage pathway consisting in the activation of ceramide synthase that catalyzes ceramide formation from sphingosine [65].

Interestingly, it has been shown that the content of ceramides is increased in the muscles and liver of obese, insulin resistant subjects, which is probably the result of an increase in the concentration of fatty acids—a substrate in de novo ceramide synthesis [36,37,38,39,40,41,42,43,44]. Moreover, the inflammatory process that accompanies obesity and the associated increased levels of proinflammatory cytokines such as tumor necrosis factor α (TNF-α) may contribute to increased ceramide levels through activation of sphingomyelinase [66,67]. In studies on 3T3-L1 adipocyte cells, it has been shown that a source of ceramide that contributes to the inhibition of GLUT4 expression is sphingomyelin hydrolysis [68]. Increased concentration of ceramides observed in serum, skeletal muscles, and liver of obese rodents and humans [69] correlates negatively with sensitivity to insulin, and positively with the circulating interleukin 6 (IL-6), which also determines the participation of ceramide in the development of insulin resistance.

However, the total ceramide content is higher not only in the muscles and liver, but also in the fat tissue of obese people compared to lean counterparts [70]. Moreover, it has been demonstrated that ceramide level in the subcutaneous tissue is higher in obese women with hepatic steatosis compared to obese individuals without this liver disease [71] and in obese T2D patients compared to obese non-diabetic patients [55,72]. Increased activity of key enzymes involved in ceramide metabolism, i.e., serine palmitoyltransferase, sphingomyelinase, and ceramidase, was observed in adipose tissue studies of obese diabetic and non-diabetic patients [73]. A strong positive correlation was also noticed between total ceramide content in the subcutaneous adipose tissue and HOMA-IR [55,60,61,70,73]. In addition, inhibition of ceramide production in adipocytes has been shown to improve insulin sensitivity [72].

Another important bioactive lipid from the sphingolipid group is sphingosine-1-phosphate (S1P). The main source of S1P is ceramide, which, in the reaction catalyzed by ceramidase, is hydrolyzed to sphingosine (Sph) and fatty acid. Then, Sph is phosphorylated to S1P by sphingosine kinases (SphKs). Two isoforms of SphK, SphK-1 and SphK-2, have been found in various tissues in mammals, including adipose tissue [74]. S1P acts via G protein-coupled receptors (S1PR1-5), the expression profile of which varies depending on the cell type [75]. This compound regulates various cellular processes that include cell growth, survival, angiogenesis, and the regulation of inflammatory processes. [75,76]. S1P has been shown to promote an inflammatory response by increasing the expression of cytokines (e.g., TNFα, IL-6, and MCP-1) in adipocytes and leading to an increase of their secretion [77]. The pro-inflammatory effect of S1P has been confirmed in studies performed on knockout mice lacking the SphK1 gene, in which reduced production of pro-inflammatory cytokines (TNFα and IL6) and decreased recruitment of adipose tissue macrophages have been observed [78]. These changes were also associated with improved insulin signaling in adipose tissue and muscles, as well as improved systemic insulin sensitivity [78]. However, it has been shown that the effect of S1P on the inflammatory response depends on the carrier protein. The major carrier proteins for S1P are apoM and albumin. Most plasma S1P is combined with apolipoprotein M (apoM)—ApoM-S1P—which preferentially binds to HDL It has been demonstrated that ApoM-S1P suppresses inflammatory responses in endothelial cells [75].

Despite the existence of data confirming that the accumulation of sphingolipids may contribute to the development of metabolic disorders, there are still very few reports on the role of this lipid group in the development of insulin resistance in adipose tissue.

## 5. Obesity, Adipokines and Inflammation

For many years, adipose tissue was considered only as an energy store. Currently, it is seen as a highly specialized tissue that plays an important endocrine function through the synthesis and secretion of many bioactive molecules—adipokines that act in adipose tissue (autocrine and paracrine activity) and affect distant organs and tissues (standard endocrine activity) [79,80]. The excess of visceral adipose tissue leads to the development of chronic inflammation, and the adipocytokines released by the visceral and subcutaneous adipose tissue into the bloodstream affect the metabolism participating in numerous metabolic processes [60,81,82,83,84,85].

In obese individuals, adverse production of adipokines is observed, which leads to the development of metabolic diseases associated with obesity. These include leptin, resistin, and lipocalin 2 as well as cytokines, such as TNF-α and IL-6. Fat tissue also produces a small group of anti-inflammatory adipokines, including adiponectin, apelin, fibroblast growth factor 21 [86,87,88,89]. The variety of adipokines and the interactions between them prove that adipose tissue affects many different metabolic processes. The importance of endocrine function of adipocytes is demonstrated with both excess of adipose tissue (overweight and obesity) and its deficiency (malnutrition and lipodystrophy) [83,90,91].

### 5.1. Adiponectin

Adiponectin is one of the few adipokines that plays an important role in the regulation of glucose and lipid metabolism. It is produced mainly and in large quantities by mature adipocytes [92]. Serum concentration of adiponectin is lower in obese people [93] and increases significantly with weight loss [94]. There is a strong positive correlation between serum concentration of adiponectin and insulin sensitivity. Weyer et al. studied the relationship between serum adiponectin concentration and glucose consumption rate measured during hyperinsulinemic clamp in a group of lean and obese people [92]. They confirmed that obesity and type 2 diabetes are associated with low plasma adiponectin levels in different ethnic groups and demonstrated that hypoadiponectinemia is more correlated with insulin resistance and hyperinsulinemia than the degree of obesity [92]. Moreover, the concentration of circulating adiponectin is more influenced by subcutaneous adipose tissue, as evidenced by the higher expression of the adipokine gene in subcutaneous adipose tissue compared to visceral tissue [95,96]. The situation is different in rodents, in which a higher expression of adiponectin is observed in the visceral adipose tissue [97]. Furthermore, animal studies have shown that administration of adiponectin increases the phosphorylation of tyrosine residues at the insulin receptor in skeletal muscles, which is associated with insulin response and effective muscle glucose uptake. In the same study, it was also observed that, in skeletal muscle samples, the administration of adiponectin to mice increased the expression of genes encoding proteins associated with the transport and oxidation of fatty acids (CD36 and acyl-CoA oxidase) as well as proteins involved in oxidative phosphorylation process, i.e., UCP (uncoupling proteins). Moreover, it has been shown that, in skeletal muscle, adiponectin not only increases the oxidation of fatty acids (AMP kinase activation), which leads to a decrease in plasma FFA concentration but also increases glucose consumption [91,98]. On the other hand, it has been shown that, in liver, adiponectin reduces the expression of the CD36 protein responsible for fatty acid transport, which may explain the beneficial effect of adiponectin on triglyceride concentration in hepatocytes [98]. Other researchers have shown that increased insulin sensitivity in the liver is caused by stimulating phosphorylation of acetyl-CoA carboxylase and reducing the inflow of non-esterified fatty acids [91,99].

In the blood vessel walls, adiponectin—in response to growth factors—inhibits the adhesion of monocytes (by lowering the expression of adhesion molecules) as well as transformation of macrophages into foam cells (by impeding the expression of scavenger receptors). Moreover, adiponectin increases nitric oxide production in endothelial cells and stimulates angiogenesis [100]. In a study on adipose tissue, Xi et al. showed that palmitate is responsible for low expression of adiponectin [101], which may be caused by the increased content of biologically active lipids containing this compound in their structure. Literature data indicate the existence of a strong negative correlation between ceramide content in adipose tissue and plasma adiponectin concentration in human [70] and animals [60,61]. Moreover, in animals, a strong negative correlation was also found between the DAG content in both subcutaneous and visceral adipose tissue and the plasma adiponectin concentration [61]. Since adiponectin levels decrease in obesity, which consequently contributes to metabolic disorders in skeletal muscle and liver, it seems that adipokine measurement may become a promising diagnostic tool for pre-diabetes. Furthermore, since the synthesis/secretion of adiponectin is at least partly dependent on the accumulation of bioactive lipids, inhibition of the synthesis of these lipids may be a therapeutic strategy in the treatment of metabolic disorders.

Although all the above-mentioned studies have shown that adiponectin has anti-diabetic, anti-inflammatory, and anti-atherosclerotic effects, further research is needed to explain the mechanism of regulation of its synthesis and release into the bloodstream.

### 5.2. Leptin

Leptin is produced by adipocytes and secreted directly into the bloodstream. It is produced in higher amounts in subcutaneous than visceral adipose tissue [102]. Moreover, the observed differences in leptin concentration are gender-dependent. In women, the values are over twice as high as in men [103]. The concentration of leptin in plasma under physiological conditions increases along with fat mass and decreases during weight loss and low-fat diet [94]. Numerous factors affect leptin expression and secretion, e.g., the growth of its content is caused by insulin, glucocorticosteroids, TNF-α, and estrogens, and decrease is affected by androgens, free fatty acids, and growth hormone [104]. It has been shown that inhibition of ceramide de novo synthesis by myriocin (SPT inhibitor) leads to a decrease in serum leptin concentration [72]. Leptin is responsible for regulating the energy balance as it acts centrally by suppressing the appetite, reducing food intake and simultaneously increasing energy expenditure, thus contributing to weight loss. Lipid metabolism may also be affected by leptin as it influences both the central and peripheral nervous system due to the presence of leptin receptors that belong to the superfamily of type I cytokine receptors. Central administration of leptin increases resting metabolic rate, which leads to a decrease in the content of triacylglycerols in both adipose and non-fat tissues, and reduces the concentration of plasma FFA and TAG in rats [104]. In the study by Schulz et al., the intranasal administration of leptin resulted in significant weight loss and fat tissue reduction in both lean rats and those with diet-induced obesity [105]. The level of leptin quickly decreases when reducing the caloric intake and during weight loss. In obese people and animals, as compared to their slim counterparts, hyperleptinemia is developed, leading to weakened response to leptin. Both endogenous high leptin levels and treatment with exogenous leptin are not effective in treating obesity due to leptin resistance [105]. The occurrence of this phenomenon may suggest that obesity is associated with leptin resistance.

### 5.3. Apelin

Apelin is a peptide that is encoded by *APLN* gene and is widely expressed in various organs and tissues such as heart, lung, kidney, liver, adipose tissue, gastrointestinal tract, and brain [106]. This peptide acts via G protein-coupled receptor APLNR [106] and has been recognized to exert different physiological effects, mainly on the cardiovascular system and on the regulation of fluid homeostasis [107]. As adipokine, apelin contributes to the regulation of glucose metabolism, lipolysis, food intake, cell proliferation, and angiogenesis [87]. Clinical studies have demonstrated a relationship between apelin concentration and obesity and insulin resistance [108]. Moreover, apelin has been shown to inhibit insulin secretion and reduce glycemia in mice [109,110]. In addition, apelin has been reported to improve insulin sensitivity during hyperinsulinemic-euglycemic clamp by increasing muscle glucose uptake [111]. It has been demonstrated that apelin-knockout mice develop insulin resistance but the adverse effects can be reversed by exogenous apelin treatment [110,111].

### 5.4. Fibroblast Growth Factor 21 (FGF21)

Fibroblast growth factor 21 is a protein belonging to a member of the fibroblast growth factor (FGF) superfamily and is encoded by the *FGF21* gene. This protein is secreted by the liver, skeletal muscles, and adipose tissue, but, regardless of the tissue from which it is secreted, it positively affects lipid and glucose homeostasis and has anti-inflammatory properties [88,89]. It has been found that FGF21 induces browning of white adipose tissue and activates brown adipocytes in response to cold exposure, thereby increasing thermogenesis. In addition, FGF21 has been shown to regulate adiponectin expression in adipocytes, and thus affects its serum levels [88]. Moreover, FGF21 acts directly on white adipocytes, inhibiting lipolysis and stimulates glucose uptake. In a clinical trial on the effects of LY2405319 (an FGF21 variant) in obese patients with T2DM, it has been demonstrated that this compound significantly improved body weight, fasting insulin, and adiponectin; decreased LDL and triglycerides; and increased HDL [112]. These promising results indicate that it is a potential pharmaceutical compound in the treatment of obesity and associated comorbidities.

Fibroblast growth factor 21 is a protein belonging to a member of the fibroblast growth factor (FGF) superfamily and is encoded by the *FGF21* gene. This protein is secreted by the liver, skeletal muscles, and adipose tissue, but, regardless of the tissue from which it is secreted, it positively affects lipid and glucose homeostasis and has anti-inflammatory properties [88,89]. It has been found that FGF21 induces browning of white adipose tissue and activates brown adipocytes in response to cold exposure, thereby increasing thermogenesis. In addition, FGF21 has been shown to regulate adiponectin expression in adipocytes, and thus affects its serum levels [88]. Moreover, FGF21 acts directly on white adipocytes, inhibiting lipolysis and stimulates glucose uptake. In a clinical trial on the effects of LY2405319 (an FGF21 variant) in obese patients with T2DM, it has been demonstrated that this compound significantly improved body weight, fasting insulin, and adiponectin; decreased LDL and triglycerides; and increased HDL [112]. These promising results indicate that it is a potential pharmaceutical compound in the treatment of obesity and associated comorbidities.

### 5.5. Lipocalin 2 (LCN-2)

Lipocalin 2 is a glycoprotein encoded by the *LCN2* gene and is expressed in several tissues including adipose tissue and liver [89]. It has been found that this protein is a component of the innate immune system that plays a key role in the acute phase response to infection. A growing body of evidence indicates that elevated plasma LCN-2 concentration is associated with inflammation, as observed in obesity and insulin resistance [113]. It has been found that the expression of this protein increases in obese diabetic mice [113], in adipocytes from obese Zucker rats [114], and in visceral adipose tissue of obese people [115]. In addition, the development of obesity is associated with changes in the extracellular matrix of adipose tissue, and the metalloproteinase enzymes (MMP-2 and MMP-9) that are actively involved in this process are activated by LCN-2 [115]. In animal models of type 2 diabetes or non-alcoholic steatohepatitis, increased LCN-2 expression promotes inflammation by recruiting inflammatory cells such as neutrophils and inducing proinflammatory cytokines [116]. Interestingly, mRNA and protein level of LCN2 in human VAT was associated with proinflammatory markers as well as with insulin levels and HOMA-IR [117].

### 5.6. Resistin

Resistin is a protein discovered relatively recently—in 2001. The place of synthesis and secretion of this hormone is species-specific. In rodents, the expression of the resistin gene occurs mainly in adipocytes [118], whereas in humans the main source of resistin are adipocytes and immune system cells: peripheral blood inflammatory cells, monocytes, macrophages, and leukocytes [119,120,121]. McTernan et al. showed that the expression level of resistin is much higher in visceral adipose tissue compared to SAT [122]. In rodents, the main role of resistin is its involvement in inducing insulin resistance. It also participates in the regulation of carbohydrate metabolism. Experiments on animals have shown that increased concentration of resistin results in reduced insulin response in insulin-sensitive tissues, and leads to glucose intolerance. This action of resistin, consisting of inhibition of insulin signaling, increased gluconeogenesis, and glycogenolysis, as well as reduction of glucose uptake, lead to increased blood glucose levels [118]. Further studies in mice conducted by Qian et al. showed that, in obese animals, serum level of resistin were significantly higher than in lean counterparts [123]. It was also found that the administration of insulin-sensitizing drugs (thiazolidinediones) inhibits resistin expression in adipose tissue and decreases its serum concentration in obese mice [124]. Analysis of human data showed that the relationship between resistin level and the development of insulin resistance is ambiguous. There are studies showing a significant correlation between resistin concentration and insulin resistance [118]; however, there are also data that do not confirm such a relationship [118]. Despite numerous studies on this issue, the results remain controversial. Although there is no clear answer about the role of resistin in the pathogenesis of insulin resistance in humans, the results of numerous experiments have shown that resistin can play a role in inflammatory processes. It was observed that resistin induces the expression of, inter alia, IL-6 and TNF-α.

Because the role of resistin in the pathogenesis of insulin resistance and type 2 diabetes in humans has not been fully elucidated, further research in this area appears to be necessary.

### 5.7. Tumor Necrosis Factor α (TNF-α)

TNF-α is a proinflammatory cytokine produced by macrophages, monocytes, adipose tissue [125], and skeletal muscles [126]. Acting in an auto- or paracrine manner, it affects the regulation of carbohydrate and lipid metabolism and is involved in the induction of insulin resistance [127]. Overexpression of TNFα, occurring, e.g., as a result of an increased supply of fatty acids to the cell, was first demonstrated in adipose tissue in obese and diabetic rodents [125]. These results have been confirmed in later studies showing that increased TNF-α production in human adipocytes is positively correlated with obesity, insulin levels, and insulin resistance [128]. In addition, elevated plasma TNFα concentration is positively correlated with increased ceramide content in human subcutaneous adipose tissue [70], as well as with both ceramide and DAG in rodent subcutaneous and visceral fat [61]. Weisberg et al. observed that the main source of TNF-α in adipose tissue of obese mice are macrophages originating from stromal vascular fraction (SVF) [129], and higher TNF-α concentration in plasma is caused by infiltration of M1 macrophages into adipose tissue. TNF-α in fat tissue inhibits the activity of enzymes connected with the metabolism of fatty acids and glucose, and reduces the secretion of some adipokines, including adiponectin [130]. Moreover, it also activates sphingomyelinases—enzymes responsible for the formation of ceramide from sphingomyelin [131]. However, the most significant mechanism by which TNF-α reduces insulin sensitivity appears to be inactivation of IRS-1. It was observed that TNF-α concentration correlates with the degree of inactivation of IRS-1 (increased phosphorylation of serine residues in IRS-1) in adipose tissue of obese patients. Such altered IRS-1 becomes an inhibitor of tyrosine kinase of the insulin receptor (IR) and impairs GLUT-4 translocation to plasma membrane, thus reducing insulin-dependent transport of glucose to the cells [132,133].

Despite clinical data demonstrating the contribution of TNF-α to the development of insulin resistance, its role is still not fully explained.

### 5.8. Interleukin 6 (IL6)

Interleukin 6 (IL-6) is a cytokine produced in the immune system cells. It participates in inflammation, defense mechanisms, and response to mechanical injuries [134]. Approximately one third of the circulating IL-6 comes from adipose tissue [135]; however, most IL-6 originating from fat tissue is secreted by the SVF consisting of endothelial cells, macrophages, and fibroblasts [94]. The synthesis and secretion of IL-6 in the visceral tissue is almost three times higher than in the subcutaneous tissue [136]. The role of IL-6 in obesity is not entirely clear. In peripheral tissues, IL-6 inhibits the expression of insulin receptors and reduces adipogenesis as well as the level of adiponectin [130,137]. Bruun et al. demonstrated that incubation of IL-6 and IL-6sR (soluble receptor for IL-6) with human adipose tissue contributes to the reduction of adiponectin mRNA level [130]. Other studies have shown that IL-6 inhibits the insulin signaling cascade, which leads to impaired phosphorylation of the insulin receptor and insulin receptor substrate 1 in hepatocytes [137]. In addition, decreased IL-6 expression in adipose tissue and reduced serum IL-6 concentration have been shown to be associated with weight loss [138]. Moreover, it has been demonstrated that plasma IL 6 concentration is positively correlated with the content of ceramides in adipose tissue [70].

## 6. Summary

Obesity is defined as an excessive accumulation of adipose tissue in the body, which contributes to overall health impairment. Obesity and the resulting health complications (insulin resistance and type 2 diabetes) are some of the main causes of mortality in developed countries. Although adipose tissue itself does not play a dominant role in insulin-dependent glucose uptake, it seems to have a paramount role in inducing systemic insulin resistance resulting from obesity. The exact mechanism by which adipose tissue induces insulin resistance is unknown; however, numerous studies have shown its significant role in this pathological process.

**Figure 1:**
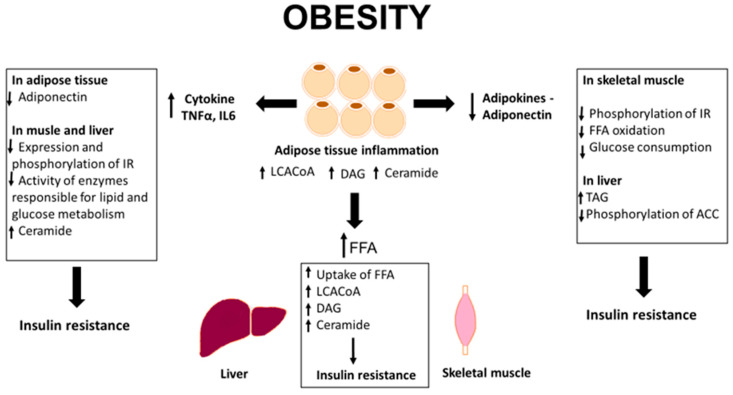
Overview of adipocytes dysfunction in obesity. IR, insulin receptor; FFA, free fatty acids; TAG, triacylglycerols; ACC, acetyl-CoA carboxylase, TNF-α, tumor necrosis factor α; IL6, interleukin 6; LCACoA, long chain acyl-CoA; DAG, diacylglycerols.

According to the latest data, the accumulation of biologically active lipids in adipose tissue is probably related to the development of insulin resistance. Studies conducted on a group of obese individuals indicated an increase in the total content of ceramide, as well as the content of DAG and LCACoA in the subcutaneous adipose tissue in obese patients compared to lean counterparts. In addition, the results obtained on the subcutaneous and epicardial adipose tissue, as well as on cell lines, indicate that these lipids contribute to the development of insulin resistance (Figure 1).

Obesity is also characterized by the co-occurrence of inflammation. Adipose tissue synthesizes and secretes numerous bioactive molecules (adipokines and cytokines) that act in an auto-, para-, and endocrine manner (Figure 1). Adiponectin is one of the few adipokines that has a protective effect on the development of metabolic disorders. All the available studies have suggested that adiponectin is a hormone with antidiabetic, anti-inflammatory, and anti-atherosclerotic effect. Another adipokine secreted by adipose tissue is leptin. It is produced by adipocytes and secreted directly into the blood. The concentration of leptin in plasma rises with increasing the adipose tissue mass and drops with decreasing the body weight and during the consumption of low-fat diet. The key role of leptin is to regulate energy balance and increase the rate of metabolism in muscles and adipose tissue. Another adipokine is resistin. Its physiological role is to maintain glycemia during starvation periods, and the pathological effect is associated with the formation of excessive fat tissue. Interleukin 6 is a pleiotropic cytokine that plays an important role in inflammatory processes, body defense mechanisms, and response to mechanical injuries. In peripheral tissues, IL-6 inhibits the expression of insulin receptors and reduces adiponectin expression, thus lowering its secretion by adipocytes. Another cytokine released by adipose tissue is TNF-α that may affect the development of insulin resistance. Increased production of TNF-α in human adipocytes is positively correlated with the degree of obesity, insulin level, and insulin resistance. Studies from recent years have shown strong correlations between the content of biologically active lipids in adipose tissue and the concentration of adipokines and cytokines in the plasma, suggesting that these lipids may be involved in the regulation of synthesis and/or secretion of adipokines and pro-inflammatory cytokines and thus contribute to the induction of insulin resistance [55,60,61,70,72,73].

Although recent studies have revealed that inflammation caused by excess adipose tissue and abnormalities in lipid metabolism may contribute to the development of obesity-related insulin resistance, the mechanism linking obesity and adipose tissue dysfunction with insulin resistance is still not fully elucidated. Understanding these relationships seems to be crucial because it could help to design new ways of early diagnosis as well as new treatments for metabolic disorders associated with obesity. A promising tool that could be useful as a marker of obesity and metabolic syndrome seems to be the circulating nucleosomes [139,140,141]. Published studies have shown that, in both humans and animals, obesity, high fat diet, and inflammation promote the release of nucleosomes into the bloodstream [139,140,141]. Recently published data have shown that circulating nucleosomes can be used as a universal marker of obesity and metabolic syndrome [142]. Although the available data are promising, further research is needed to determine the exact mechanism for inducing insulin resistance associated with obesity.

## Abbreviations

ACS	Acyl-CoA synthetase
ADA	American Diabetes Association
BMI	Body mass index
Cer	Ceramide
DAG	Diacylglycerols
DGAT	diacylglycerol acyltransferase
FABPpm	fatty acid-binding protein plasma membrane
FATP	fatty acid transport proteins
FFA	Free fatty acids
GPAT	glycerol-3-phosphate acyltransferase
HDL	high-density lipoprotein
HSL	hormone-sensitive lipase
IR	insulin receptor
IRS-1	insulin receptor substrate-1
LCACoA	long-chain acyl-CoA
LDL	low-density lipoprotein
LPA	lysophosphatidic acid
LPL	lipoprotein lipase
PA	phosphatidic acid
PAP	phosphatidic acid phosphatase
PI3K	phosphoinositide 3-kinases
PKA	Protein kinase A
PKC	protein kinase C
SAT	subcutaneous adipose tissue
SPT	serine palmitoyltransferase
SVF	stromal vascular fraction
TAG	Triacylglycerols
TNF-α	tumor necrosis factor-α
VAT	visceral adipose tissue
VLDL	very low-density lipoproteins
